# The secret of self-fertilizing plants: *NIN*-*NAD1*'s role in symbiotic nitrogen fixation

**DOI:** 10.1093/plcell/koae237

**Published:** 2024-08-21

**Authors:** Min-Yao Jhu, Jian Feng

**Affiliations:** Assistant Features Editor, The Plant Cell, American Society of Plant Biologists; Crop Science Centre, Department of Plant Sciences, University of Cambridge, Cambridge CB30LE, UK; Key Laboratory of Seed Innovation, Institute of Genetics and Developmental Biology, Chinese Academy of Sciences, Beijing 100101, China; University of Chinese Academy of Sciences, Beijing 101408, China; CAS-JIC Centre of Excellence for Plant and Microbial Science (CEPAMS), Institute of Genetics and Developmental Biology, Chinese Academy of Sciences, Beijing 100101, China

Imagine if crops could naturally fertilize themselves, eliminating the need for synthetic fertilizers and revolutionizing agriculture. This possibility hinges on some plants’ ability to engage in nitrogen-fixing symbiosis with bacteria (Jhu and Oldroyd 2023; Huisman and Geurts 2019). Symbiotic nitrogen fixation (SNF) is primarily found in the nitrogen-fixing clade (NFC) of plants, which includes species from the orders Fabales, Cucurbitales, Fagales, and Rosales (Huisman and Geurts 2019). So far, SNF has 2 forms: rhizobial symbiosis in legumes and actinorhizal symbiosis in various nonlegume NFC species. Both types originated from a common ancestor over 100 million years ago and involve housing bacteria within specialized organs called nodules (Werner et al. 2014). Genomic analyses suggest a predisposition for nodulation in the NFC's common ancestor (Griesmann et al. 2018; Zhang et al. 2024), but direct genetic evidence remains sparse. The central question in the field is: What are the genetic and molecular mechanisms underlying SNF in NFC plants?

Previous research on rhizobial symbiosis in legumes identified around 200 genes involved in nodulation (Roy et al. 2020). The SNF mechanism likely arose from modifications to existing genes rather than the acquisition of new ones (Liu et al. 2022). Among these critical genes, *NODULE INCEPTION* (*NIN*) is essential for rhizobial infection and nodule development by regulating multiple symbiosis-responsive genes (Griesmann et al. 2018; Zhang et al. 2024; Shen and Feng 2024). In addition to *NIN*, *Nodules with Activated Defense1* (*NAD1*) is crucial for rhizobial accommodation in symbiotic cells (Wang et al. 2016). Mutation of *NAD1* causes necrotic nodules and loss of nitrogen fixation without affecting infection thread formation or nodule development (Wang et al. 2016). However, it is unclear if NIN also regulates genes involved in bacterial intracellular accommodation. In a new study, **Haixiang Yu and colleagues (Yu et al. 2024)** investigated the genetic and molecular mechanisms underlying SNF in NFC plants, focusing on the *NIN–NAD1* regulatory module. Through whole-genome alignment of *NAD1* and its homologs across species, they confirmed that *NAD1* is exclusive to NFC plants, present in both nodulating and non-nodulating species within this clade but absent in the outgroups (Figure). The presence of *NAD1* in non-nodulating NFC plants suggests it may have predated nodulation, initially serving a different role that later adapted to symbiosis (Yu et al. 2024).

**Figure.**
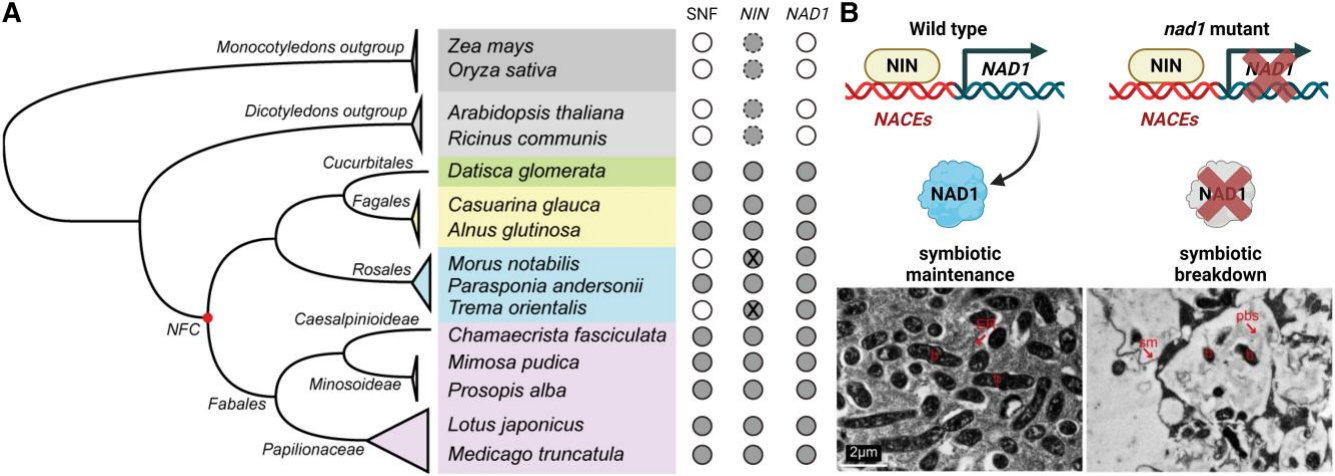
The role of *NAD1* in bacterial intracellular accommodation in NFC plant nodules. **A)** Phylogenetic tree of NFC plants, showing their relationships with monocot and dicot outgroups. The presence or absence of SNF, *NIN*, and *NAD1* is indicated by circles next to the tree: white (absent), gray (present), gray with dotted lines (NIN-like proteins but not NIN orthologs present), and gray with “X” (premature stop codons in *NIN*). **B)** Diagram illustrating how NIN binds to NACEs to regulate *NAD1* expression, facilitating bacterial intracellular accommodation during symbiosome development. Transmission electron microscopy (TEM) images show symbiosomes (sm), bacteroids (b), and the peribacteroid space (pbs) in wild-type *M. truncatula* and *nad1* mutants. Scale bars, 2 *μ*m. This figure was adapted and combined from Figs. 5 and 6 of Yu et al. (2024) and created using BioRender.com.

Using transcriptome data, reverse transcription-quantitative polymerase chain reaction (RT-qPCR) analysis, and GUS reporter assays, Yu et al. demonstrated that *NAD1* is specifically expressed in the nodules of NFC plants. Additionally, *NAD1* expression is regulated by conserved nodulation-associated cis-regulatory elements (NACEs) in the *NAD1* promoter. These elements are bound by the NIN transcription factor, the master regulator in nodulation (Shen and Feng 2024). NIN directly regulates *NAD1* by binding to these NACEs (Figure), ensuring nodule-specific expression (Yu et al. 2024). The *NIN–NAD1* module may represent a key evolutionary development in the acquisition of nodulation in NFC plants.

In legumes like *Medicago truncatula* and *Lotus japonicus*, loss of *NAD1* resulted in defective nodules with impaired nitrogen fixation and premature senescence. Many legumes accommodate rhizobia in membrane-bound compartments within nodule cells known as symbiosomes (Jhu and Oldroyd 2023). These symbiosomes differentiate into nitrogen-fixing bacteroids (Jhu and Oldroyd 2023). Transmission electron microscopy revealed abnormal symbiosomes and bacteroid degradation in *nad1* mutants (Figure), further confirming *NAD1*'s role in maintaining symbiosome integrity and function during nodule development. Furthermore, functional complementation experiments showed that *NAD1* promoters containing NACEs from different NFC species, including actinorhizal plants, could restore the nodulation defects in legume *nad1* mutants (Yu et al. 2024). This underscores the conserved nature of these regulatory elements and their evolutionary significance.

The identification of *NAD1* and its regulatory elements not only answers key evolutionary questions but also opens new avenues for agricultural innovation. By harnessing the regulatory mechanisms of *NAD1* and *NIN*, scientists can potentially engineer nitrogen-fixing capabilities in nonlegume crops, helping to reduce our reliance on synthetic fertilizers, promote sustainable agricultural practices, and ensure food security.
